# Real-time molecular optical micro-imaging of EGFR mutations using a fluorescent erlotinib based tracer

**DOI:** 10.1186/s12890-018-0760-z

**Published:** 2019-01-07

**Authors:** Maxime Patout, Florian Guisier, Xavier Brune, Pierre Bohn, Anthony Romieu, Nasrin Sarafan-Vasseur, Richard Sesboüé, Pierre-Yves Renard, Luc Thiberville, Mathieu Salaün

**Affiliations:** 1 Rouen University Hospital, Clinique Pneumologique & CIC INSERM U 1404, F-76000 Rouen, France; 2 Normandie University, UNIROUEN, LITIS, Quant.I.F – EA 4108, F-76000 Rouen, France; 3 Normandie University, COBRA, UMR 6014 & FR 3038; CNRS, F-76000 Rouen, France; 4 Institut de Chimie Moléculaire de l’Université de Bourgogne, UMR 6302, CNRS, University, Bourgogne Franche-Comté, 21078 Dijon, France; 5 Génétique du cancer et des maladies neuropsychiatriques, Normandie University, UNIROUEN, INSERM, F-76000 Rouen, France; 6 Service de Pneumologie, Oncologie Thoracique, Soins Intensifs Respiratoires, CHU de Rouen, 1 rue de Germont, 76031 Rouen Cedex, France

**Keywords:** Epidermal growth factor, *EGFR*, Molecular imaging, Lung cancer, Fibred confocal fluorescence microscopy, Erlotinib, Theranostic

## Abstract

**Background:**

*EGFR* mutations are routinely explored in lung adenocarcinoma by sequencing tumoral DNA. The aim of this study was to evaluate a fluorescent-labelled erlotinib based theranostic agent for the molecular imaging of mutated *EGFR* tumours in vitro and ex vivo using a mice xenograft model and fibred confocal fluorescence microscopy (FCFM).

**Methods:**

The fluorescent tracer was synthesized in our laboratory by addition of fluorescein to an erlotinib molecule. Three human adenocarcinoma cell lines with mutated *EGFR* (HCC827, H1975 and H1650) and one with wild-type *EGFR* (A549) were xenografted on 35 Nude mice. MTT viability assay was performed after exposure to our tracer. In vitro imaging was performed at 1 μM tracer solution, and ex vivo imaging was performed on fresh tumours excised from mice and exposed to a 1 μM tracer solution in PBS for 1 h. Real-time molecular imaging was performed using FCFM and median fluorescence intensity (MFI) was recorded for each experiment.

**Results:**

MTT viability assay confirmed that addition of fluorescein to erlotinib did not suppress the cytotoxic of erlotinib on tumoral cells. In vitro FCFM imaging showed that our tracer was able to distinguish cell lines with mutated *EGFR* from those lines with wild-type *EGFR* (*p* < 0.001). Ex vivo FCFM imaging of xenografts with mutated *EGFR* had a significantly higher MFI than wild-type (*p* < 0.001). At a cut-off value of 354 Arbitrary Units, MFI of our tracer had a sensitivity of 100% and a specificity of 96.3% for identifying mutated *EGFR* tumours.

**Conclusion:**

Real time molecular imaging using fluorescent erlotinib is able to identify ex vivo tumours with *EGFR* mutations.

**Electronic supplementary material:**

The online version of this article (10.1186/s12890-018-0760-z) contains supplementary material, which is available to authorized users.

## Background

Lung cancer is the most frequent cause of cancer related death in the world [1]. Lung adenocarcinoma is the most common histological subtype [2]. In Europe, 17.3% of lung adenocarcinoma tumours harbour a mutation of the epidermal growth factor receptor (EGFR) [3]. EGFR is a transmembrane tyrosine kinase receptor that promotes cell proliferation and survival [4]. De novo activating mutations occurring on the intracellular tyrosine kinase activity domain of *EGFR* can drive tumour growth [4]. Targeted tyrosine kinase inhibitor (TKI) therapies such as gefitinib or erlotinib inhibit EGFR activation by competitive inhibition at the ATP binding site. As first-line treatment, these TKI improve progression-free survival in patients with tumours harbouring *EGFR* sensitive mutations [5]. Therefore, identification of patients with tumours harbouring such mutations is now recommended to guide first line treatment.

To identify these patients, the gold standard technique is DNA sequencing of *EGFR* on tumoral material. In France, this analysis is performed in regional oncologic molecular platforms for all patients with lung adenocarcinoma. This approach allows identification of all eligible patients but has dramatically increased the workload of pathologists. Several techniques have been developed to test for *EGFR* mutations [6]. As most of these techniques are expensive, diagnostic algorithm and nomogram have been proposed to rationalize their use [7, 8].

Theranostic agents can be defined as therapeutic agent used for diagnostic purposes. They are of increased interest in oncology [9] and could be an innovative way to rationalize the use of *EGFR* analysis technics. To date, most of the published theranostic agents targeting *EGFR* have used full body imaging such as Positon Emission Tomography (PET) [10–21] or magnetic resonance imaging (MRI) [22, 23]. These imaging techniques can provide useful information on the bio-distribution of theranostic agents but expose patients to radiations and to a drug that may have systemic effects. Furthermore, these imaging techniques cannot provide a sufficient resolution for cellular imaging.

Fibred confocal fluorescent microscopy (FCFM) is a non-invasive imaging technique that can provide real-time in vivo microscopic imaging during a bronchoscopy [24–26]. FCFM can be used with fluorescent tracers in several pathologic conditions such as invasive aspergillosis using a fluorescent specific peptide [27]. In oncology, fluorescent agents have been described for the assessment of tumour response [28] or for the imaging of human EGFR 2 [29]. For EGFR imaging, fluorescent monoclonal antibodies have been used in colorectal cancer to assess *EGFR* expression [30, 31]. However, these antibodies target the extracellular domain of the EGFR and are not able to identify *EGFR* mutations. As erlotinib and gefitinib bind to the intracellular domain of mutated EGFR, radio-labelled erlotinib and gefitinib have been assessed to image *EGFR* mutated tumours [15, 16, 20, 21]. To our knowledge, no study has assessed the feasibility of using fluorescence-labelled EGFR TKI as a theranostic agent in order to perform real time molecular imaging of *EGFR*-mutated tumours.

The aim of this study was to evaluate a fluorescent-labelled erlotinib-based tracer for the molecular imaging of *EGFR*-mutated tumours in vitro and ex vivo using a mice xenograft model and FCFM imaging.

## Methods

### Cell lines

We used four human tumoral cell lines obtained from the American Type Culture Collection (ATTC). Cells lines were chosen according to their *EGFR* status and their sensitivity to erlotinib. HCC827 cell line, which harbours E746_A750 mutation on the exon 19 of *EGFR*, is hypersensitive to erlotinib and has more than 20 copies of the mutated *EGFR* gene [32]. H1650 cell line, which harbours the DelE746_A750 mutation on the exon 19 of *EGFR*, is insensitive to erlotinib because of persistent activation of the p-ten pathway [33] and has 4 copies of the *EGFR*-mutated gene [34]. A549 cell line, with wild-type *EGFR*, is resistant to erlotinib and has 2.5 copies of the *EGFR* gene [35]. H1975 cell line harbours two EGFR mutations: T790 M, which confers resistance to erlotinib [36] and L858R, which confers sensitivity to erlotinib. H1975 has from 2.8 to 6.2 copies of the *EGFR* gene [34, 35] and is insensitive to erlotinib. HCC827, H1650, H1975 were cultured in RPMI (with 10% foetal calf serum) and A549 cells were cultured in DMEM (with 10% foetal calf serum) according to ATTC recommendations. All cells were grown at 37 °C in an atmosphere of 5% CO2.

### Animal model

All animal experiments were approved by Rouen University animal research committee (approval n° 0312–01). Forty-eight 4-weeks-old female Swiss Nude Nu/Nu mice were used for the study. Animals were commercially acquired to Charles Rivers Laboratories, Saint-Germain-sur-L’Arbersle, France. Animals were kept in an approved laboratory animal facility. They were housed in a ventilated cage with a room temperature set at 25 °C, a humidity set between 45 and 49% and 12-h light and obscurity alternation. Each cage contained a maximum of 5 animals. Animals had unrestricted access to food and water. Cages were cleaned weekly. All animal experiments were performed under sterile conditions. Xenografts were performed using 1 × 10^6^ of exponentially growing tumoral cells. Tumoral cells were removed from the plate with 2 ml 0.05% EDTA trypsin and suspended in phosphate-buffered saline (PBS). Subcutaneous injection of tumoral cells was performed in mice’s flank under general anaesthesia induced by inhalation of 2% isoflurane. Xenografts were grown until they reached a size of 2 mm. To allow 8 mice to be analysed in each group, we hypothesize that at least two-third of xenografted mice would survive until sufficient tumoral growth.

### EGFR mutated fluorescent tracer

**Fig. 1:**
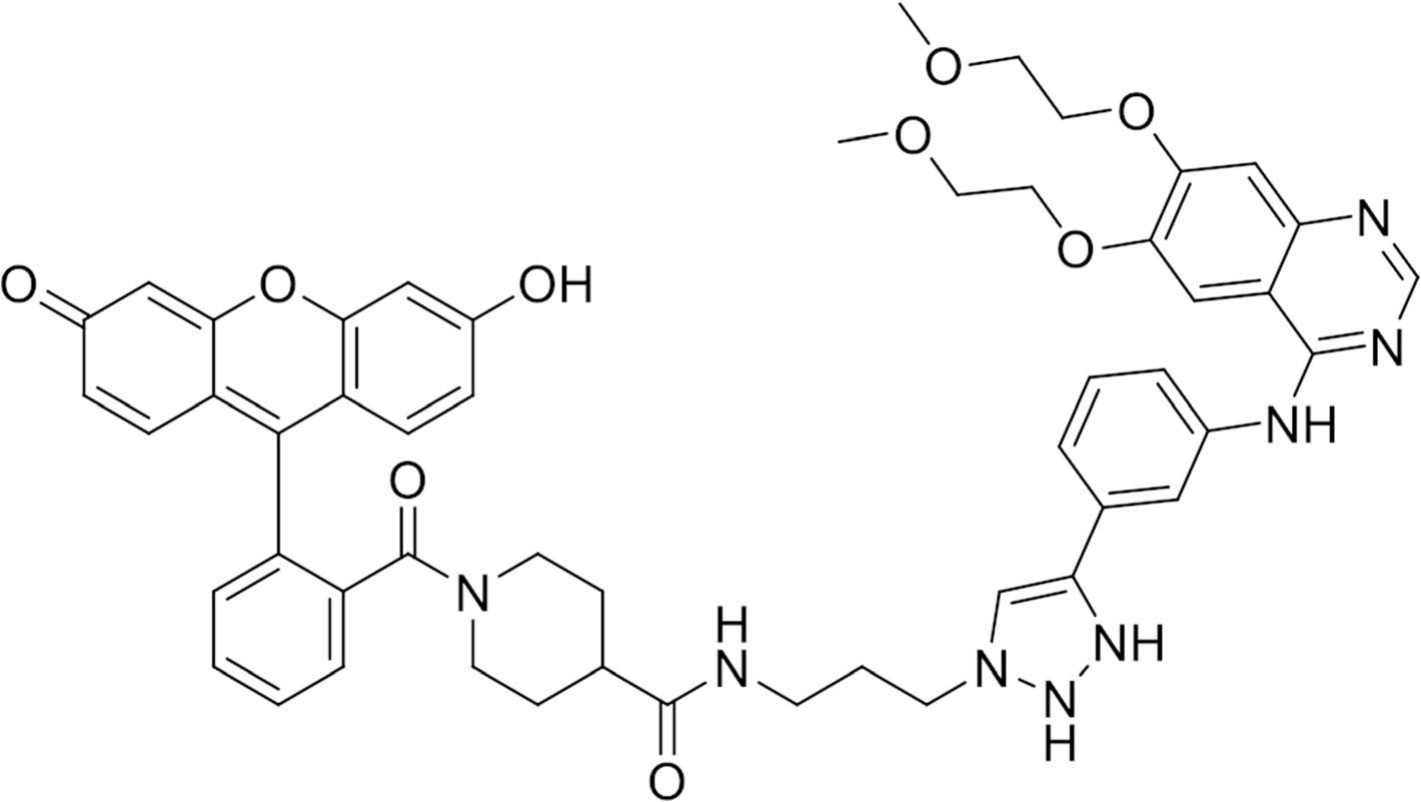
Fluorescent erlotinib based tracer

Tracer was designed and produced at the COBRA UMR CNRS 6014 laboratory, Rouen University, France. The tracer was obtained by adding fluorescein to erlotinib using a linker (Fig. 1). The tracer had a molar mass of 1374.37 g/mol. Its maximum fluorescence excitation wavelength was 507 nm and the maximal emission wavelength was 528 nm. Full details of our tracer synthesis can be found in Additional file 1.

### Imaging

FCFM imaging was performed using a Cellvizio lab® system (Mauna Kea Technologies, France). The 488 nm laser light source illuminates the tissue through an optical fibre probe that is applied onto the tumour surface. The same probe and laser source were used throughout the study. The system captures fluorescent signal emitted in a wavelength range from 500 to 620 nm. The probe had a diameter of 1.4 mm corresponding to a 603 μm circular field of view. Lateral and axial resolution of the system were 5 μm and 15 μm respectively. Before each imaging procedure, the probe was calibrated using dedicated Cellvizio® tool, according to the manufacturer recommendations. During the imaging procedure, the optical probe was recalibrated every 30 min. to avoid spontaneous auto-fluorescence noise. For each experiment, real-time imaging was captured at a frame rate of 8 to 12 images/second using the CellVizio v1.2.0 dedicated software (Mauna Kea Technologies, Paris, France). Images were obtained by direct application of the probe tip onto the specimen. Recorded images were analysed using the CellVizio Viewer 1.6 (Mauna Kea Technologies, Paris, France). Each recorded sequence was analysed, frame-by-frame, with lower and upper level thresholds of the look-up table set to 1 and 8000 expressed in arbitrary unit (AU). For each image, the median intensity was recorded. For each sequence, the image with the maximal median fluorescence intensity (MFI) was selected. MFI was calculated by CellVizio Viewer Signal Quantification toolbox that ranged from 1 to 8000 AU.

### In vitro experiments

Cell viability after exposure to the tracer was assessed using 3-[4,5-diméthylthiazol-2yl]-2,5-diphényltétrazolium bromide test (MTT) (CellTiter96® non-radioactive cell proliferation assay (Promega, Madison, USA). Cell viability was assessed at increased concentration of tracer and genuine erlotinib. As our tracer had a 3.5-fold higher molar mass than erlotinib, cell viability assay was also performed at equimolar erlotinib concentrations. For each cell line, we calculated the concentration that resulted in 50% cell death (IC50). MTT assay was chosen because of its lack of interference with the emission wavelength of our tracer.

In vitro imaging was performed on centrifuged cell pellet. Cells were removed from the plates at confluence by trypsination (1 ml of Trypsin in 0.05% EDTA), suspended in 8 ml of culture medium, centrifuged twice at 2000 rpm for 3 min and finally re-suspended in PBS at room temperature. 1 × 10^6^ cells were re-suspended in a non-fluorescent culture container using 0.5 ml of PBS containing 1 μmol/l of tracer, 1 μmol/l of erlotinib or PBS alone during an hour at 37 °C, in the dark, with 5% CO2 atmosphere. Cells were then washed twice using PBS. Each washing was followed by 3 min centrifugation at 2000 rpm. FCFM imaging was then immediately performed by application of the probe on the cell pellet. Experiments and imaging were performed six times for each cell line. Results of imaging are expressed as median fluorescence intensity (MFI), in arbitrary units (A.U).

### Ex vivo experiments

For ex vivo imaging, xenografts were excised under general anaesthesia after intra-peritoneal injection of 100 mg/kg of ketamine and 10 mg/kg of xylazine. After xenograft excision, and under general anaesthesia, mice were sacrificed by cervical dislocation. The xenograft was then immerged in a 2 ml solution containing 1 μmol/l of tracer at room temperature and kept out of light. After an hour, each tumour was washed by consecutive immersion in 3 separate wells containing 2 ml of 0.9% saline serum. FCFM imaging was then immediately performed by application of the optical probe on the surface of each xenograft. Experiments and imaging were performed at least eight times for each cell line. Results of imaging are expressed as MFI.

### Statistics

Results are expressed as headcounts and percentages, medians, and first and third quartiles (IQR) or means and standard deviation (SD). Comparisons were performed using Student T-test and ANOVA test. Correlations were assessed using the Spearman correlation coefficient. ROC-curve was used to establish sensitivity and specificity and to determine optimal cut-off value. All tests were two-sided, the type I error rate was set at 0.05. Analyses were performed using GraphPad Prism 6® for Mac OS X® (GraphPad Software, La Jolla, CA, USA).

## Results

### Cell viability assay

**Fig. 2:**
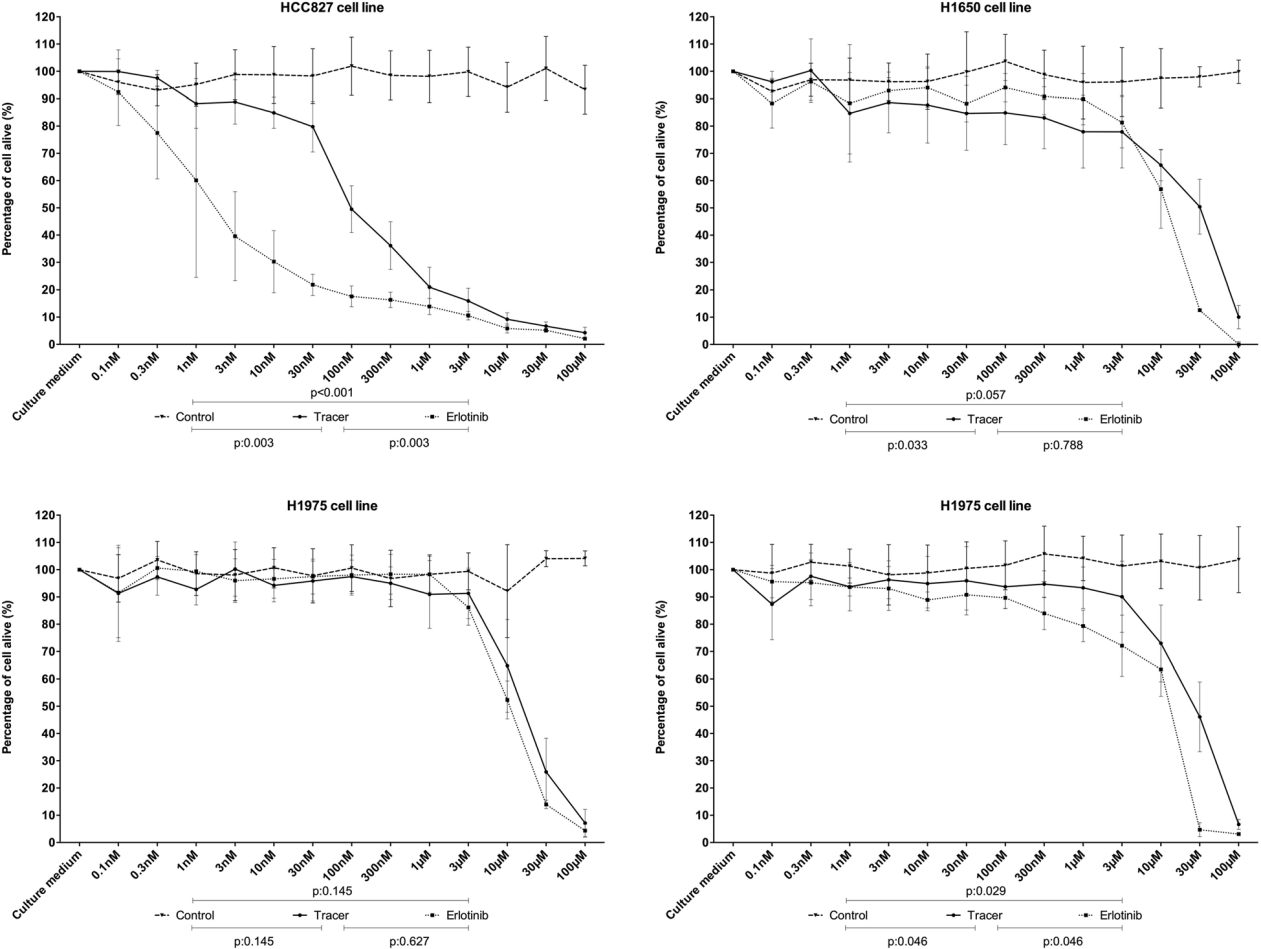
MTT viability assay for each cell line to increasing concentrations of erlotinib, tracer and DMSO. Results are expressed in percentage of cell alive to increasing concentrations of tracer, erlotinib and control (DMSO) (ns = not significant)

Cell viability assay confirmed the sensitivity profile to erlotinib for each cell line. HCC827 was the only cell line sensitive to erlotinib. Cell viability assay showed that the erlotinib fluorescent tracer and erlotinib did not differed significantly regarding their IC50 which was 100 nmol/l in both cases (*p* = 0.1) (Fig. 2).

### In vitro imaging

**Fig. 3:**
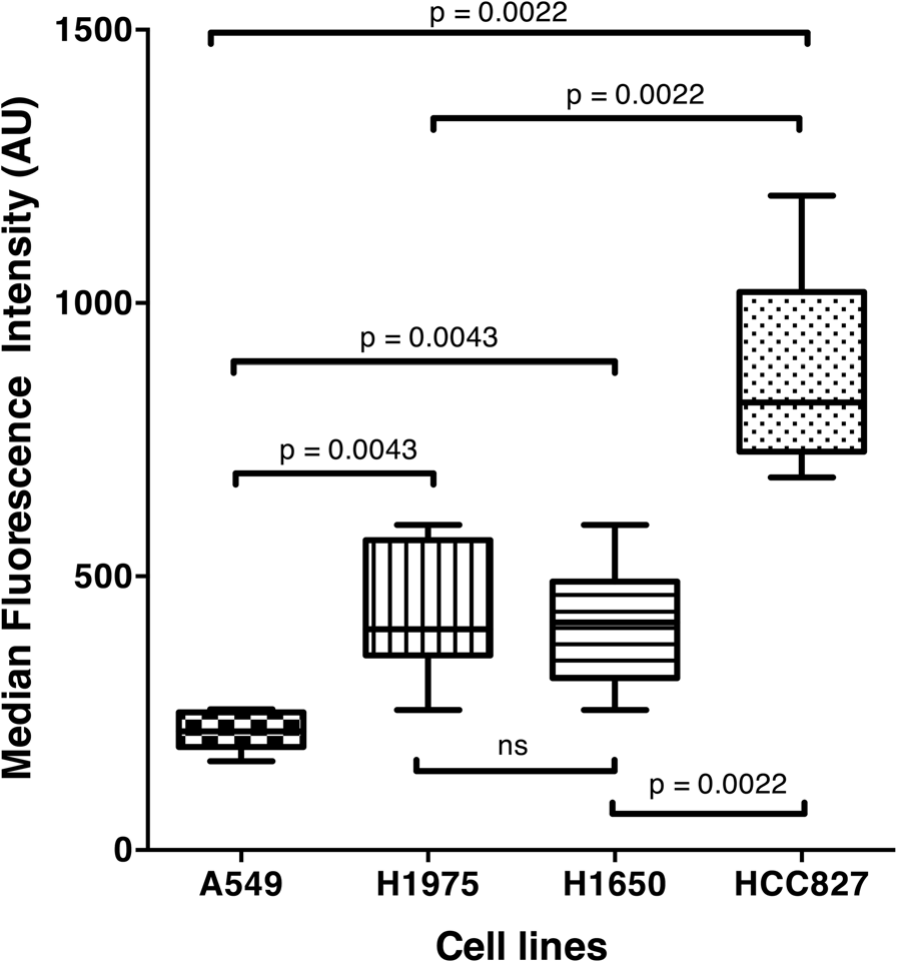
Median Intensity recorded by FCFM imaging following 1-h contact between cell pellet and tracer at a concentration of 1 μmol/l for each cell line: A549, H1975, H1650 and HCC827 (*p* < 0.001) (ns = not significant)

FCFM imaging of cell lines exposed to erlotinib alone did not show any spontaneous fluorescence. MFI obtained during FCFM imaging of cell pellets after exposure to 1 μmol/l of tracer for an hour differed in each tumoral cell line, and was significantly higher in HCC827 cells (MFI = 819 AU (728–1020] vs. MFI = 217 AU [188–251] in A549; MFI = 404 AU [356–566] in H1975; and MFI = 417 AU [315–491] in H1650; *p* < 0.001). The tracer was able to distinguish cell lines with a mutated *EFGR* from wild-type *EGFR* cell lines, and to distinguish cell lines with a sensitive phenotype (HCC827) from those with a resistant phenotype (H1975 and H1650) (*p* = 0.002) (Fig. 3).

### Ex vivo imaging

**Fig. 4:**
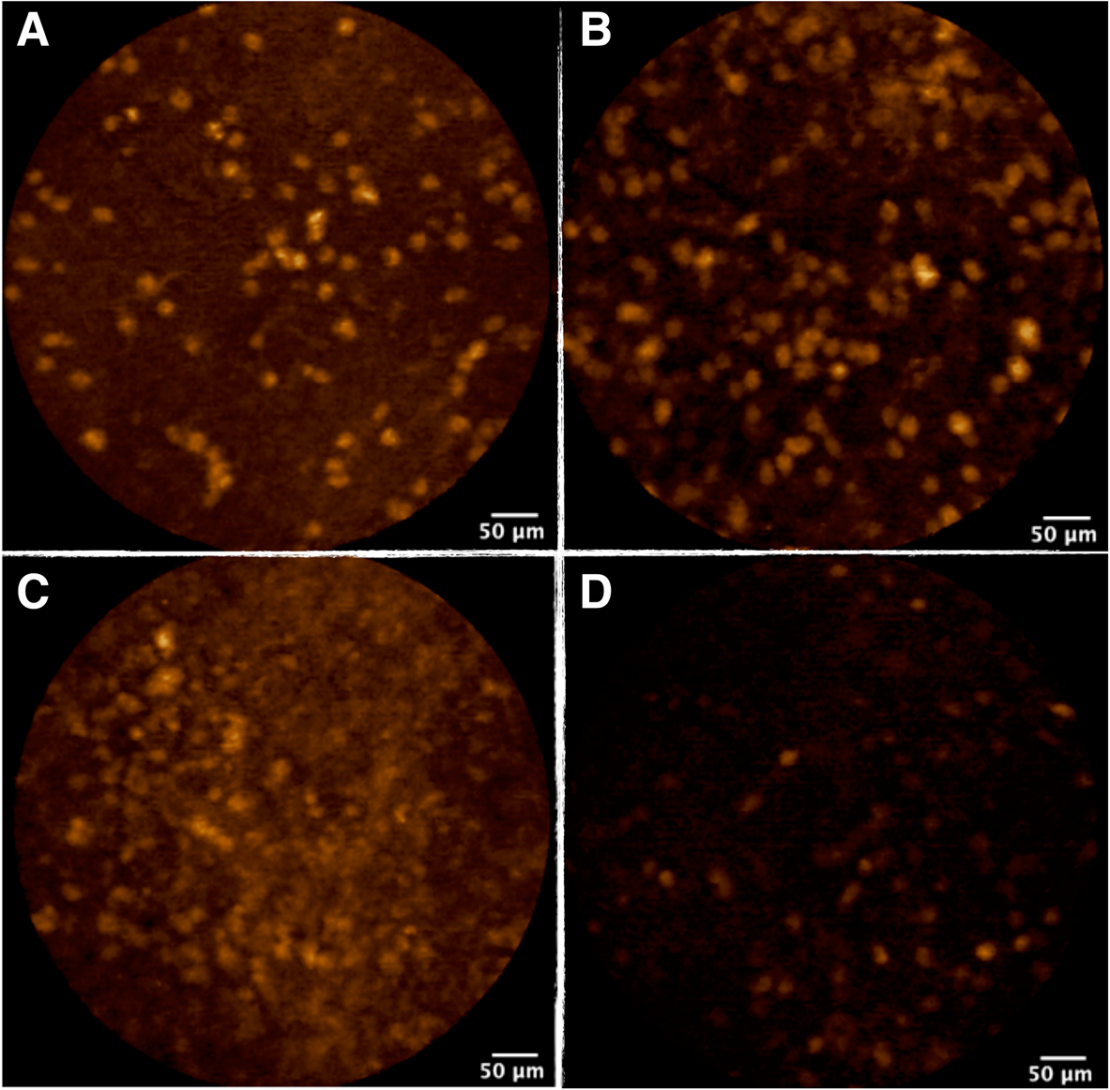
FCFM imaging from ex vivo xenograft following 1-h immersion in tracer at a concentration of 10 μmol/l. Image A: HCC827 line - Image B: H1650 line - Image C: H1975 line - Image D: A549 line

FCFM imaging on excised tumour was performed on 8 HCC827, on 8 A549, on 8 H1975, and on 11 H1650 xenografts. FCFM imaging of xenografts performed before immersion in the tracer solution showed no spontaneous fluorescence (Fig. 4).

**Fig. 5:**
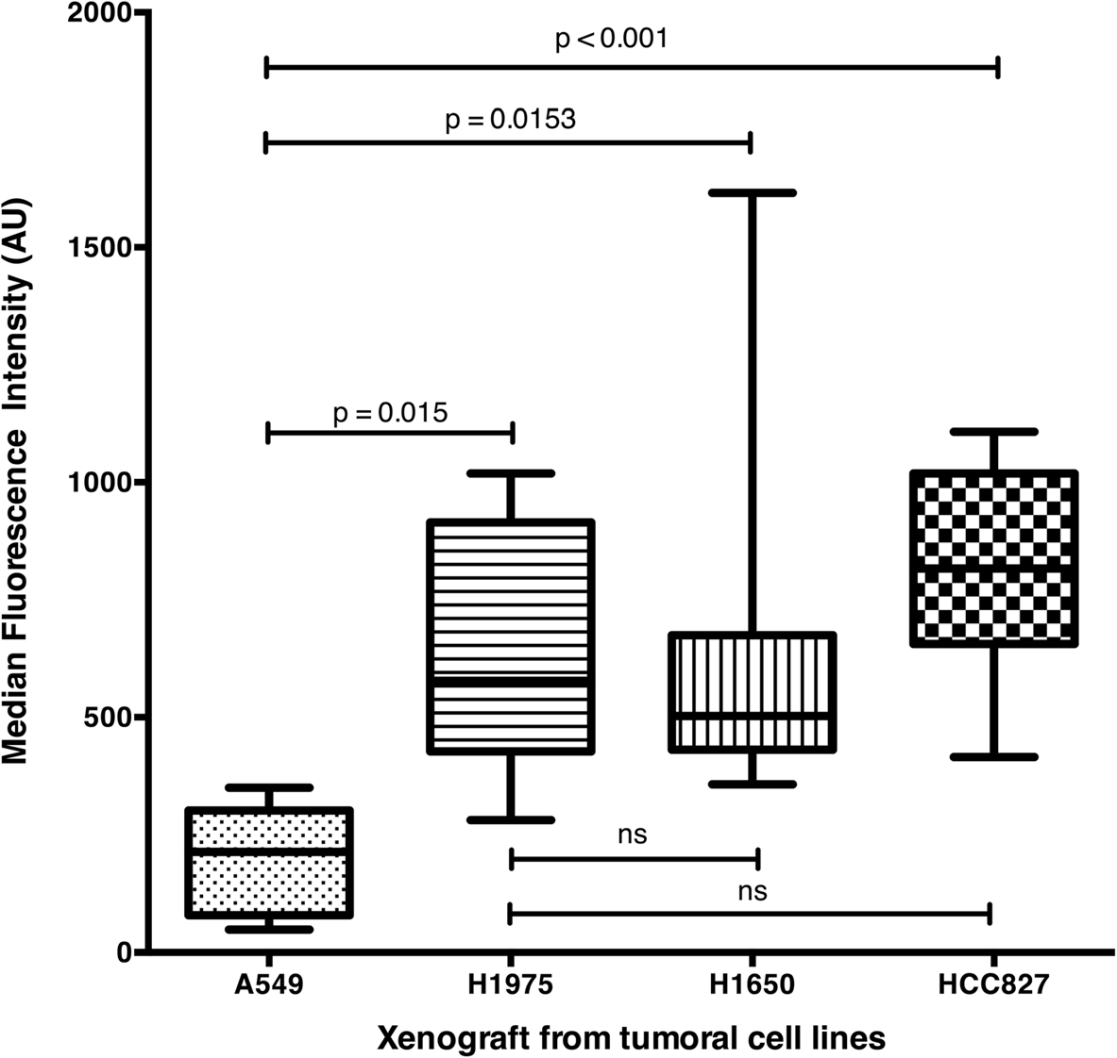
Median Fluorescence Intensity recorded by FCFM imaging following 1-h immersion in a solution of tracer at 1 μmol/l for xenografts derived from cell line HCC827, H1650, A549 and H1975 (*p* < 0.001)

MFI was significantly lower in xenografts derived from A549 cell line, which harbours a wild-type *EGFR*: 214 A.U [78–302] when compared to the ones derived from HCC827 cell line: A.U = 818 [657–1019], H1650 cell line: A.U = 503 [432–675] and H1975 cell line: A.U = 578 [428–915], (*p* < 0.001, *p* = 0.013, *p* = 0.015 respectively). Using a cut-off value of 354 A.U, MFI had a sensitivity of 100% and a specificity of 96.3% for identifying mutated *EGFR* tumours. MFI did not differ significantly between xenografts derived from the three cell lines with a mutation of *EGFR* (HCC827, H1650 and H1975). Therefore, ex vivo imaging could not distinguish tumours with a sensitive phenotype from those with a resistant phenotype to erlotinib (Fig. 5).

## Discussion

This study shows for the first time that fluorescent erlotinib can be used as a tracer to distinguish lung adenocarcinoma cells with *EGFR* mutation from wild-type EGFR, in real time, both in vitro and ex vivo. The ex vivo technique had a sensitivity of 100% and specificity of 96% for the diagnosis of *EGFR* mutation. In this study, use of labelled erlotinib as an ex vivo fluorescent tracer did not allow to differentiate *EGFR* mutated cells that are sensitive to erlotinib, from the resistant ones.

In this study, the cytotoxic activity of erlotinib was not altered by the adjunction of the fluorescein residue despite modifications of the molar mass and of the steric effect of the molecule. The preserved cytotoxic activity of the tracer addresses one potential limitation, as no standard confocal microscopy was performed to confirm the intracellular localization of the tracer. The functional consequences of the adjunction of the fluorescein residue were not assessed in the study. They may have a significant impact in the activity of the erlotinib. Despite such impact, our tracer kept a cytotoxic activity. This is important to ascertain the theranostic feature of this erlotinib-based tracer. Furthermore, previous studies on theranostic erlotinib only assessed the EGFR phosphorylation following exposure to the tracer and not its cytotoxic effect [14, 15, 20, 37].

In the in vitro experiments, the tracer was able to distinguish tumoral cells with a wild-type *EGFR* from those with a mutated *EGFR*. Likewise, the tracer was able to distinguish tumoral cells with a sensitive phenotype from those with a resistant one. This result has to be interpreted cautiously. Firstly, this difference in fluorescence intensity could be partially explained by the number of *EGFR* copies that each cell lines carry. Indeed, H1650 and H1975 have less than 5 copies of *EGFR* [34, 35] whereas as HCC827 has more than 20 copies [32]. Yet, unlike in colorectal cancer, the number of *EGFR* copies has no clinical or therapeutic consequence on the management of lung cancer. Therefore, this result should not preclude the use of this tracer in lung adenocarcinomas. Secondly, the theranostic agent presented in this study only inhibits the *EGFR* pathway whereas other pathways can be involved in tumour growth. For instance, the H1650 cell line has an abnormal activation of p-ten pathway that induces tumour growth and confers resistance to erlotinib [33] despite the effective inhibition of the *EGFR* pathway. To address this limitation, the combination with other published theranostic molecules targeting could be interesting [28, 38, 39]. Thirdly, other mutations, such as T790 M mutation, present in the H1975 cell line, may have modified the binding of this tracer to EGFR. Indeed, T790 M mutation increases EGFR affinity to ATP which acts as a competing inhibitor of TKI [36]. A way to address this limitation would be to develop a tracer using third generation TKI that are effective to stop tumour growth in patients harbouring T790 M mutations [40–42].

In this study, the tracer was not able to distinguish tumours harbouring the T790 M resistance mutation from the other *EGFR*-mutated cells, but its presence is rare in TKI-naïve patients [43, 44]. If the T790 M mutation is the most common acquired resistance mechanism to *EGFR* specific TKI [45, 46], other mechanisms of resistance such as MET amplification can occur [45, 46]. Hence, for patients with tumoral progression under TKI treatment, bronchial biopsies should be repeated in order to identify the resistance mechanism and to give an adequate second line treatment. As EFGR mutations are not found in normal tissue, positivity of the ex vivo test would confirm that the bronchial sample contains tumoral cells. This rapid and easy to perform ex vivo imaging process may improve the diagnostic yield of bronchoscopy. In such situation, the presence of fluorescence following ex vivo use of tracer would guarantee to the physician that the biopsy taken is an adequate tumoral sample and not necrotic tissue. As the technique avoids any systemic administration of a modified licensed drug to the patient, any risk of adverse event for the patient is eluded.

However, we did not perform any test on human bronchial samples in that study. Thus, we are not able to assess any deleterious consequence of the immersion of the biopsy in the tracer. Similarly, the MFI cut-off value chosen to diagnose mutated EGFR tumors may be different in human bronchial samples as their spontaneous fluorescence may create background noise.

In conclusion, the use of this tracer was associated with a high sensitivity to detect swiftly mutated EGFR tumour. Because it wouldn’t have to be administered to patients, we believe that this fluorescent tracer could be used as a pre-test diagnostic tool to confirm that the sampling of the tumour was adequate in patient with progressive disease under EGFR specific TKI therapy. These experimental results require further confirmation studies on fresh human bronchial biopsies.

## Conclusions

Our study describes a novel fluorescent theranostic molecule that can perform ex vivo for molecular imaging of EGFR mutations and identify mutated form of EGFR. Further studies using human tumours are required to evaluate the usefulness of this tracer as a diagnostic tool of EGFR mutations.

## Additional file

Additional file 1: Tracer synthesis. Chemical steps to produce the tracer used in our experiments. (DOCX 474 kb)

## Abbreviations

ATTC: American Type Culture Collection
AU: Arbitrary Unit
EGFR: Epidermal growth factor
FCFM: Fibred confocal fluorescent microscopy
IC50: Concentration that resulted in 50% cell death
IQR: Interquartile
MFI: Median fluorescence intensity
MRI: Magnetic resonance imaging
MTT: 3-[4,5-diméthylthiazol-2yl]-2,5-diphényltétrazolium bromide test
PBS: Phosphate-buffered saline
PET: Positon Emission Tomography
ROC: Receiving operator curve
SD: Standard deviation
TKI: Tyrosine kinase inhibitor
