# Supplementary material for: Real-time molecular optical micro-imaging of EGFR mutations using a fluorescent erlotinib based tracer
Source: BMC Pulm Med. 2019 Jan 7;19:3. doi: 10.1186/s12890-018-0760-z (PMC6322267; doi:10.1186/s12890-018-0760-z)
Supplement: Supplementary file 1 — Tracer synthesis. Chemical steps to produce the tracer used in our experiments. (DOCX 474 kb) [file 12890_2018_760_MOESM1_ESM.docx]

**Annex 1 – Tracer synthesis**

Synthesis was conducted by Xavier Brune under supervision of Profs. Romieu and Renard.

***Synthesis of XB01:***

To a cold solution of isonipecotic acid (1.0 g, 7.75 mmol, 1 equiv) in water (10 mL) at 0 °C was added K_2_CO_3_ (2.5 g, 18 mmol, 2.3 equiv) followed by a dropwise addition of benzyl chloroformate (1.3 mL, 9.1 mmol, 1.15 equiv). After 4 h of stirring, the reaction mixture was washed with ethyl acetate (20 mL). The aq. layer was acidified with HCl 10% (10 mL) and extracted twice with ethyl acetate (2 x 20mL). The organic layers were combined and dried over MgSO_4_. Solvents were evaporated under reduced pressure and compound was obtained as a white solid (1.94 g, 95%). ^1^H NMR (300 MHz, CDCl_3_) δ = 1.22 (q, ^3^*J* = 4.2Hz, 2H) ; 1.90 (q, ^3^*J* = 4.2Hz, 2H) ; 2.52 (m, 1H) ; 2.94 (t, ^3^*J* = 11Hz, 2H) ; 4.1 (m, 2H) ; 5;12 (s, 2H) ; 7.35 (m, 5H), corresponding to the analyses made by Dr. S. Dautrey.

***Synthesis of XB02:***

 To a solution of **XB01** (0.9 g, 3.4 mmol, 1 equiv) in *t*BuOH (40 mL). 4-Dimethylaminopyridine (125 mg, 1.02 mmol, 0.3 equiv) was added followed by the addition of Boc_2_O (1.5 g, 6. 8mmol, 2 equiv). The reaction mixture became yellow and began gassing. The reaction was stirred overnight. The residue was dissolved in ethyl acetate (20 mL) and then washed by HCl 1N (20 mL), aq. 5% NaHCO_3_ (20 mL) and a brine (20 mL). Solvents were evaporated under reduced pressure. Colourless oil was obtained (0.97 g, 88%). ^1^H NMR (300 MHz, CDCl_3_) δ = 1.22(q, ^3^*J* = 4.2Hz, 2H) ; 1.45 (s, 9H) ; 1.92(q, ^3^*J* = 4.2Hz, 2H) ; 2.52 (m, 1H) ; 2.97 (t, ^3^*J* = 11Hz, 2H) ; 4.1 (m, 2H) ; 5.12 (s, 2H) ; 7.35 (m, 5H) , corresponding to the analyses made by Dr. S. Dautrey.

***Synthesis of XB03:***

 To a solution of **XB02** (0.779g, 2.44mmol) in ethanol (20mL) was added Palladium activated on carbon 10% (0.1g) under a dihydrogen atmosphere. The reaction stirred overnight. A filtration using Celite was done and solvent was evaporated under reduce pressure. White solid was obtained (0.427g, 94%). ^1^H NMR (300 MHz, CDCl_3_) δ = 1.24(q, ^3^*J* = 4.2Hz, 2H); 1.46 (s, 9H); 1.90(q, ^3^*J* = 4.2Hz, 2H); 2.56 (m, 1H); 2.93 (t, ^3^*J* = 11Hz, 2H); 4.2 (m, 2H), corresponding to the analyses made by Dr. S. Dautrey.

***Synthesis of XB04:***

To a solution of fluorescein (0.200 g, 0.6 mmol, 1 equiv) and **XB03** (0.150 g, 0.81 mmol, 1.35 equiv) in dry DMF (2 mL) was added *N*,*N*'-dicyclohexylcarbodiimide (0.140 g, 0.68 mmol, 1.13 equiv), Hydroxybenzotriazole monohydrate (0.098 g, 0.72 mmol, 1.2 equiv) and finally *N*,*N*-diisopropylethylamine (100 µL, 0.6 mmol, 1 equiv). The reaction was heated at 70 °C overnight. Solvent was removed under reduced pressure and purification was performed with a flash purification system Biotage (system B). Colourless oil was obtained (0.150 g, 50%). All spectroscopic data are identical to those reported by Massif et al^[[1]](#footnote-1)^.

***Synthesis of XB05:***

To a solution of **XB04** (0.100 g, 0.2 mmol, 1 equiv) in Dichloromethane (50 mL) was added trifluoroacetic acid (15 mL). The reaction stirred overnight. Solvent was removed under reduced pressure with a water aspirator and purification on reversed-phase has been made assisted by biotage (system B). White solid was obtained (0.084 g, 95%). All spectroscopic data are identical to those reported by Massif et al2^0^.

***Synthesis of XB06:***

To a solution of 1-bromo-3-aminopropane (3 g, 0.014 mol, 1 equiv) in water (30 mL) was added sodium azide (3 g, 0.46 mol, 3.05 equiv). The reaction stirred overnight at reflux. One third of the water was removed under reduced pressure. At 0 °C, ethylacetate (30 mL) and potassium hydroxide in pellets (2 g) was added. The aqueous layer was washed twice with Ethylacetate (20 mL). The organic layers were dried over MgSO_4_. Solvent was removed under reduced pressure. Colourless oil was obtained (0.926 g, 68%). ^1^H NMR (300 MHz, CDCl_3_) δ = 1.1(s, 2H); 1.73 (dt, ^3^*J* = 6.8Hz, 2H); 2.81 (t, ^3^*J* = 6.8Hz, 2H); 3.37 (t, ^3^*J* = 6.8Hz, 2H)

***Synthesis of XB07:***

To a solution of **XB05** (0.1 g, 0.23 mmol, 1 equiv) in DMF (11 mL) was added **XB06** (0.023 g, 0.23 mmol, 1 equiv), N,N-Diisopropylethylamine (42 µL, 0.25 mmol, 1.1 equiv) and Benzotriazol-1-yloxy-tris(dimethylamino)-phosphonium hexafluorophosphate (0.120 g, 0.27 mmol, 1.2 equiv). The reaction stirred for 48 h. Solvent was removed under reduced pressure and purification by reversed-phase HPLC (system A) was made. Red solid was obtained (0.108 mg, 91 %). All spectroscopic data are identical to those reported by Massif et al2^0^

Coupling reaction between erlotinib and derivated fluorescein:

To a solution of derivated fluorescein (30 mg, 57 µmol, 1 equiv) in DMSO (2 mL) and water (0.6 mL) was added Erlotinib (33 mg, 85 µmol, 1.5 equiv), sodium ascorbate (11 mg, 55 µmol, 0.96 equiv) and then copper(II) sulfate (11 mg, 44 µmol, 0.8 equiv) under inert atmosphere. The reaction stirred overnight. Solvent was removed under reduced pressure with a rotary vane pump and purification by reversed-phase HPLC (system A) was made. Yellow amorphous powder was obtained (31 mg, 60%).

ESI+ [M+H]+= 918.37.

Synthesis of XB09:

To a solution of 7-hydroxy-4-methyl-2H-chromen-2-one (2 g, 11 mmol, 1 equiv) in water (20 mL) was added formaldehyde (4.5 mL, 120 mmol, 11 equiv) and potassium carbonate (1.55 g, 11 mmol, 1 equiv). The mixture was heated at 50 °C for 2 h. The solution was acidified with HCl 10% and stirred for an hour at 0 °C. Then the mixture was filtered. After purification by biotage a white solid was obtained (1.25 g, 55%). ^1^H NMR (300 MHz, DMSO-d_6_) δ = 2.40 (s, 3H) ; 4.27 (br, 1H) ; 4.66 (s, 2H) ; 6.18 (s, 1H) ; 6.90 (d, ^3^*J* = 8.7Hz, 1H) ; 7.58 (d, ^3^*J* = 8.7Hz, 1H) ; 10.53 (s, 1H) ^13^C NMR (50MHz, CDCl_3_) : δ = 19.4 ; 55.3 ; 112.5 ; 113.1 ; 113.3 ; 125.2 ; 127.1 ; 151.0 ; 153.3 ; 160.9 ESI+ [M+H]+= 207

Synthesis of XB10:

To a solution of **XB09** (100 mg, 485 µmol, 1 equiv) in dichloromethane (18 mL) was added triethylamine (0.13 mL, 970 µmol, 2 equiv). Methanesulfonyl chloride (0.07 mL, 873 µmol, 1.8 equiv) was added dropwise at 0 °C. The solution was stirred 2 h at room temperature. Then water (20 mL) was added to the solution. The layers were separated and the aq. layer was washed with dichloromethane (2 x 20 mL). The organic layers were dried over MgSO_4_. Solvent was removed under reduced pressure. Purification was made by column chromatography (AcOEt/Cyclohexane; 1/1). A white solid was obtained (60 mg, 44%). ^1^H NMR (300 MHz, DMSO-d_6_) δ = 2.48(s,3H) ; 3.53(s, 3H) ; 4.68 (s, 2H) ; 5.25 (s, 1H) ; 6.47 (s, 1H) ; 7.41 (d, ^3^*J* = 8.8Hz, 1H) ; 7.84 (d, ^3^*J* = 8.8Hz, 1H) ^13^C NMR (50MHz, CDCl_3_) : δ = 19.6 ; 38.4 ; 57.3 ; 112.1 ; 114.1 ; 114.4 ; 126.3 ; 126.8 ; 151.9 ; 156.3 ; 160.9 ESI+ [M+H]+= 285.29

***Synthesis of XB11:***

To a solution of **XB09** (500 mg, 2.3 mmol, 1 equiv) in dichloromethane (12 mL) was added triethylamine (0.22 mL, 1.6 mmol, 0.66 equiv). P-Toluenesulfonyl chloride (320 mg, 1.6 mmol, 0.66 equiv) was added dropwise at 0 °C. The solution was stirred 2 h at room temperature. Then water (20 mL) was added to the solution. The layers were separated and the aqueous layer was washed with dichloromethane (2 x 20 mL). The organic layers were dried over MgSO_4_. Solvent was removed under reduced pressure. Purification was made by column chromatography (AcOEt/Cyclohexane; 1/1). White solid was obtained (340mg, 40%). ^1^H NMR (300 MHz, CDCl_3_) δ = 2.43(s, 3H) ; 2.49(s, 3H) ; 4.74 (s, 2H) ; 6.31 (s, 1H)  ; 7.06 (d, ^3^*J* = 8.7Hz, 1H) ; 7.38 (d, ^3^*J* = 8.1Hz, 2H) ; 7.52 (d, ^3^*J* = 8.7Hz, 1H) ; 7.81 (d, ^3^*J* = 8.1Hz, 1H) ^13^C NMR (50MHz, CDCl_3_) : δ = 19.1 ; 21.4 ; 57.1 ; 112.1 ; 112.4 ; 112.8 ; 123.3 ; 124.6 ; 126.3 ; 126.8 ; 130.3 ; 130.4 ; 140.3 ; 140.5 ; 149.1 ; 151.9 ; 156.3 ; 160.9 ESI+ [M+H]+= 361.38

***Synthesis of XB12:***

To a solution of **XB09** (100 mg, 0.49 mmol, 1 equiv) in dichloromethane (10 mL) was added pyridine (0.2 mL, 2.5 mmol, 5 equiv). Phosgene (5 mL, 1 mmol, 2 equiv) was added dropwise at 0 °C under argon atmosphere. The solid that appeared was filtered and was recrystallised in pentane. White solid was obtained (8mg, 7%). ^1^H NMR (300 MHz, CDCl_3_) δ = 2.43(s, 3H); 4.71 (s, 2H) ; 6.31 (s, 1H)  ; 7.38 (d, ^3^*J* = 8Hz, 2H) ; 7.81 (d, ^3^*J* = 8Hz, 1H) ^13^C NMR (50MHz, CDCl_3_) : δ = 19.4 ; 67.3 ; 111.6 ; 112.2 ; 112.3 ; 125.2 ; 129.3 ; 148.4 ; 149.1 ; 151.6 ; 160.3 ESI+ [M+H]+= 233.19

***Synthesis of XB15:***

A solution of 3-aminophenol (1 g, 9.16 mmol, 1.6 equiv) in ethyl acetate (5 mL) was heated at 80 °C for 30 min. Then ethyl chloroformate (0.53 mL, 5.6 mmol, 1 equiv) was added dropwise. The solution was stirred for an hour and was cooled down to room temperature. The mixture was filtered and the solid was washed with ethyl acetate (3 x 20 mL) and petroleum ether (3 x 20 mL). Solvents were removed under reduced pressure. Brown crystals were obtained (1 g, quantitative). ^1^H NMR (300 MHz, DMSO-d_6_) δ = 1.22 (t, ^3^*J* = 7Hz, 3H) ; 4.09 (q, ^3^*J* = 7Hz, 2H) ; 6.36 (t, ^3^*J* = 8Hz, 1H) ; 6.83 (d,  ^3^*J* = 8Hz, 1H) ; 6.85 (s, 1H) ; 7.00 (d, ^3^*J* = 8Hz, 1H) ; 9.30 (s, 1H) ; 9.45 (s, 1H) ^13^C NMR (50MHz, CDCl_3_) : δ = 12.9 ; 62.1 ; 102.5 ; 114.1 ; 114.3 ; 131.2 ; 137.8 ; 158.0 ; 163.3 ESI+ [M+H]+= 182.19

***Synthesis of XB16:***

 To a solution of 3-hydroxyphenylcarbamate (500 mg, 2.3 mmol, 1 equiv) in H_2_SO_4_ (7 mL) was added ethyl acetoacetate (0.45 mL, 3.5 mmol, 1.25 equiv). The solution was stirred for 4 h at room temperature. The mixture was then poured into cold water (30 mL). The solution was filtered and was crystallized from absolute ethanol (30 mL). White solid was obtained (400 mg, 58%). ^1^H NMR (300 MHz, DMSO-d_6_) δ = 1.26 (t, ^3^*J* = 7Hz, 2H) ; 2.38 (s, 3H) ; 4.16 (q, ^3^*J* = 7Hz, 2H) ; 6.22 (s, 1H) ; 7.40 (dd, ^3^*J* = 8.6Hz, 2H) ; 7.55 (s, 1H) ; 7.69 (d, ^3^*J* = 8.6Hz, 1H) ; 10.13 (s, 1H) ^13^C NMR (50MHz, CDCl_3_) : δ = 13.2 ; 19.2 ; 62.5 ; 112.5 ; 115.1 ; 118.6 ; 119.3 ; 125.7 ; 137.8 ; 153.3 ; 158.0 ; 160.4 ; 163.3 ESI+ [M+H]+= 248.25

***Synthesis of XB17:***

 To a solution of 4-methyl-2-oxo-2H-chromen-7-ylcarbamate (100 mg, 0.4 mmol, 1 equiv) in methanesulfonic acid (28 mL) was added paraformaldehyde (1.8 g). The solution was stirred for 8 h at 80 °C. The mixture was then poured into cold water (30 mL) and the solution was neutralized by slow addition of 10 N NaOH. The aq. phase was extracted with ethyl acetate (3 x 20 mL). The organic phases were collected and washed with ultrapure water (4 x 20 mL) and finally with brine (20 mL). The organic layer was dried over MgSO_4_ and concentrated under reduced pressure until a precipitate appeared. The solution was filtered. Brown powder was obtained (65 mg, 70%). ^1^H NMR (300 MHz, DMSO-d_6_) δ = 2.38(s, 3H) ; 3.34(s, 2H) ; 7.40 (dd, ^3^*J* = 8.6Hz, 2H) ; 7.55 (s, 1H) ; 7.69 (d, ^3^*J* = 8.7Hz, 1H) ^13^C NMR (50MHz, CDCl_3_) : δ = 19.4 ; 62.3 ; 105.3 ; 112.5 ; 115.1 ; 123.3 ; 125.8 ; 133.2 ; 148.3 ; 153.4 ; 157.7 ; 160.4 ESI+ [M+H]+= 232.20

Synthesis of XB22:

 To a solution of **XB17** (50 mg, 0.21 mmol, 1 equiv) in acetonitrile (1 mL) was added potassium fluoride (12 mg, 0.21 mmol, 1 equiv) and cryptand [2,2,2] (95 mg, 0.21 mmol, 1.2 equiv). The solution was stirred for 8 h at room temperature. Solvent was removed under reduced pressure. Purification was made by column chromatography (AcOEt/Cyclohexane; 1/1). Light yellow solid was obtained. ^1^H NMR (300 MHz, CDCl_3_) δ = 2.33(s, 3H); 5.93 (s, 1H); 6.13 (s, 2H); 6.59 (dd, ^3^*J* = 8.67Hz, 1H) ; 7.43 (d, ^3^*J* = 8.67Hz, 1H) ^13^C NMR (50MHz, CDCl_3_) : δ = 18.3 ; 75.1 ; 110.1 ; 112.3 ; 112.8 ; 117.0 ; 125.2 ; 145.8 ; 150.1 ; 152.7 ; 160.2 ^19^F NMR (282.5MHz, CDCl_3_) δ = −80.3  ESI+ [M+H]+= 208.20

***Synthesis of XB18:***

To a solution of resorcinol (2 g, 18.2 mmol, 1 equiv) in sulfuric acid conc. (20 mL) was added ethyl 4-chloroacetoacetate (2.48 mL, 18.2mmol, 1 equiv). The solution was stirred for 16 h at room temperature. The mixture was then poured into cold water (400 mL). The precipitate was filtered and washed with cold water. The solid is filtered on silica pad with ethyl acetate. The solvent is removed under reduced pressure. White powder was obtained (3.4 g, 89%). ^1^H NMR (300 MHz, DMSO-d_6_) δ = 4.91 (s, 2H) ; 6.25 (s, 1H) ; 6.57 (d, *J* = 2.4Hz, 1H) ; 6.70 (dd, *J* = 8.8Hz, 2.4Hz, 1H) ; 7.57 (d, ^3^*J* = 8.8Hz, 1H) ^13^C NMR (50MHz, CDCl_3_) : δ = 54.6 ; 103.1 ; 112.1 ; 112.7 ; 114.8 ; 127.0 ; 155.1 ; 155.6 ; 158.1 ; 160.2 ESI+ [M+H]+= 211.61

***Synthesis of XB19:***

A solution of **XB18** (100 mg, 0.4 mmol, 1 equiv) in water (80 mL) and DMF (10 mL) was refluxed for 24 h. The solvent is removed under reduced pressure. White powder was obtained (3.7 g, 93%). ^1^H NMR (300 MHz, DMSO-d_6_) δ = 4.91 (s, 2H) ; 6.25 (s, 1H) ; 6.57 (d, *J* = 2.4Hz, 1H) ; 6.70 (dd, *J* = 8.8Hz, 2.4Hz, 1H) ; 7.57 (d, ^3^*J* = 8.8Hz, 1H) ^13^C NMR (50MHz, CDCl_3_) : δ = 64.3 ; 103.4 ; 112.2 ; 112.3 ; 114.9 ; 127.6 ; 143.4 ; 155.3 ; 158.2 ; 160.3 ESI+ [M+H]+= 193.17

***Synthesis of XB20:***

To a solution of **XB19** (120 mg, 0.6 mmol, 1 equiv) in dichloromethane (5 mL) was added 4-Toluenesulfonyl chloride (120 mg, 0.6 mmol, 1 equiv) and triethylamine (0.1 mL, 0.6 mmol, 1 equiv) at 0 °C. The solution was then stirred for 2 h at room temperature. The solvent was removed under reduced pressure. The product was pure at the end of the reaction. White powder was obtained (200 mg, quantitative). ^1^H NMR (300 MHz, DMSO-d_6_) δ = 2.43 (s, 3H) ; 4.71 (d, ^3^*J* = 5.5Hz, 2H) ; 5.67 (t, ^3^*J* = 5.5Hz, 1H) ; 6.46 (s, 1H) ; 7.01 (dd, ^3^*J* = 6.6Hz, 1H) ; 7.05 (s, 1H) ; 7.49 (d, ^3^*J* = 8.8Hz, 2H) ; 7.72 (d, ^3^*J* = 8.8Hz, 1H) ; 7.80 (d, ^3^*J* = 6.6Hz, 2H) ^13^C NMR (50MHz, CDCl_3_) : δ = 24.1 ; 70.4 ; 102.2 ; 110.3 ; 112.3 ; 114.9 ; 124.7 ; 124.8 ; 127.6 ; 130.4 ; 131.0 ; 140.3 ; 140.6 ; 147.4 ; 155.6 ; 158.1 ; 160.5 ESI+ [M+H]+= 347.35

Synthesis of XB21:

To a solution of **XB20** (90 mg, 0.26 mmol, 1 equiv) in acetonitrile (5 mL) was added potassium fluoride (15 mg, 0.26 mmol, 1 equiv) and cryptand [2,2,2] (120 mg, 0.32 mmol, 1.2 equiv). The solution was stirred 8 h. Solvent was removed under reduced pressure. Purification was made by column chromatography. Light yellow powder was obtained (25 mg, 49%). ^1^H NMR (300 MHz, DMSO-d_6_) δ = 4.93 (s, 2H) ; 6.24 (s, 1H) ; 6.56 (d, *J* = 2.4Hz, 1H) ; 6.70 (dd, *J* = 8.8Hz, 2.4Hz, 1H) ; 7.57 (d, ^3^*J* = 8.8Hz, 1H) ^13^C NMR (50MHz, CDCl_3_) : δ = 94.4 ; 102.8 ; 112.3 ; 113.2 ; 117.9 ; 125.8 ; 155.2 ; 157.3 ; 159.9 ; 160.1 ^19^F NMR (282.5MHz, CDCl_3_) δ = −104.3  ESI+ [M+H]+= 195.16

1. Massif, C.; Dautrey, S.; Haefele, A.; Ziessel, R.; Renard, P.-Y.; Romieu, A. *Org. Biomol. Chem.* **2012**, *10*, 4330. [↑](#footnote-ref-1)
